# Clinical significance of genetic alterations in endoscopically obtained pancreatic cancer specimens

**DOI:** 10.1002/cam4.3723

**Published:** 2021-01-16

**Authors:** Shinichi Takano, Mitsuharu Fukasawa, Hiroko Shindo, Ei Takahashi, Sumio Hirose, Yoshimitsu Fukasawa, Satoshi Kawakami, Hiroshi Hayakawa, Natsuhiko Kuratomi, Makoto Kadokura, Shinya Maekawa, Tadashi Sato, Nobuyuki Enomoto

**Affiliations:** ^1^ First Department of Internal Medicine Faculty of Medicine University of Yamanashi Chuo Japan

**Keywords:** EUS‐FNA, FFPE, microsatellite instability, next‐generation sequencing, pancreatic cancer

## Abstract

Although comprehensive gene analyses of pancreatic cancer provide new knowledge on molecular mechanisms, the usefulness and possibility of the analyses in routinely available clinical samples remain unclear. We assessed the possibility and utility of target sequencing of endoscopically obtained pancreatic cancer samples. Fifty‐eight pancreatic cancer patients who underwent EUS‐FNA or endoscopic biopsy were enrolled. The extracted DNA quantity was assessed and used for next‐generation sequencing (NGS) of 50 cancer‐related genes from which gene mutations, copy number alterations, and microsatellite instability (MSI) were extracted via secondary analysis. A median of 19.2 ng (3.8–228) of DNA was extracted from formalin‐fixed paraffin‐embedded samples. Gene alterations were detected in 55 of 58 samples (94.8%), including all samples with a DNA concentration below the detection limit (*n* = 11). Four frequently altered genes were *KRAS* (83%), *TP53* (66%), *SMAD4* (26%), and *PTEN* (17%), and molecular targetable genes were detected in 13 cases (22.4%). Five samples (8.6%) had many mutations and suspected MSI with impaired mismatch repair genes. A Cox regression analysis revealed that metastasis (*p* < 0.005, hazard ratio [HR] 10.1), serum CEA >5 ng/ml (*p* = 0.01, HR 2.86), ≤10 detected hotspot mutations (*p* = 0.03, HR 9.86), and intact Ras signaling (*p* < 0.005, HR 5.57) were associated with a poor pancreatic cancer prognosis. We performed small, targeted sequencing of pancreatic cancer using available samples from real clinical practice and determined the relationship between gene alterations and prognosis to help determine treatment choices.

## Graphical abstract

We performed next‐generation sequencing of endoscopically obtained formalin‐fixed paraffin‐embedded tissue samples from actual clinical samples of patients with pancreatic cancer to evaluate gene alterations and potential prognostic indicators. This targeted sequencing of cancer‐related genes was informative for predicting prognosis and selecting therapy.

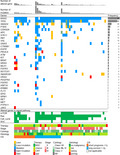

## 1 INTRODUCTION

Pancreatic cancer is a dismal disease with a 5‐year survival rate of less than 5% in the United States^1^ and 4.7% in Japan.^2^ The poor prognosis is due to the difficulty in diagnosing pancreatic cancer in the early stages and the lack of efficient therapies. Recently, new therapies for unresectable tumors such as molecular targeted therapies and immunotherapies have been gaining attention because of their different mechanisms compared with conventional anticancer agents^3^ and their efficacy in some tumor types; however, few molecular targeted drugs or immune checkpoint inhibitors are available for pancreatic cancer treatment because these drugs are only effective in a minority of pancreatic cancer patients. Therefore, precision medicine that provides the best therapy for an individual patient with pancreatic cancer according to the genetic profile of the tumor is essential.

Recently, whole‐genome or whole‐exome sequence analyses of pancreatic cancer have been conducted using next‐generation sequencing technology (NGS) and have revealed various types of genetic alterations including chromosomal rearrangements, focal amplifications, and mutations and deletions in many genes including previously reported four main genes: *KRAS*, *TP53*, *CDKN2A*, and *SMAD4*.^4^, ^5^ These comprehensive analyses have uncovered the genetic landscape of pancreatic cancer and clarified its subtypes^5^ and genetic evolution; however, the clinical utility of these analyses has been limited because they often use resected samples instead of clinically available biopsy samples, and whole‐exome or whole‐genome sequencing analyses require too much time for clinical decisions. Moreover, it is difficult to obtain sufficient sequencing depth in tumor cell‐poor tissues from clinically available samples.

Endoscopic ultrasound‐guided fine needle aspiration (EUS‐FNA) or endoscopic biopsy of an invading tumor are the primary methods of obtaining tumor tissues from pancreatic cancer; at times, these methods result in insufficient samples. Recent advances in PCR methods using high fidelity DNA polymerases and NGS have enabled rapid, accurate, and comprehensive gene analyses that can detect multiple gene mutations and copy number variations (CNVs) simultaneously and with high sensitivity, even with low amounts of DNA from clinical samples, such as formalin‐fixed paraffin‐embedded (FFPE) tissues.^6^, ^7^, ^8^

In this study, we performed NGS on endoscopically obtained FFPE samples from patients with pancreatic cancer to identify therapeutic targets and determine the clinical significance of these targets through comparisons with the clinical information of the patients.

## 2 MATERIALS AND METHODS

### 2.1 Patients and tissue samples

We retrospectively reviewed the medical records of 58 patients with pancreatic cancer who underwent EUS‐FNA or endoscopic biopsy of an invading tumor at the Yamanashi University hospital between July 2014 and February 2018. In four cases that were diagnosed as pathologically negative by EUS‐FNA, pancreatic cancer was established based on other cytological tests, imaging, and their malignant clinical course (Table S1). Further, we excluded two cases wherein NGS could not be performed due technical errors that prevented histological assessment of patients scheduled for surgery. Tissue samples were obtained as 8‐μm‐thick sections derived from one or two FFPE blocks and tumor components were separated from these sections using a Laser Capture Microdissection System (LCM, ArcturusXT, Life Technologies). DNA extraction from the LCM specimens was performed as previously reported.^6^, ^7^ DNA from the biopsied specimens was extracted using GeneRead DNA FFPE Kits (QIAGEN, Hilden, Germany) according to the manufacturer's specifications. The quantities and qualities of the extracted DNA were assessed by a NanoDrop instrument (Thermo Fisher) and the Qubit platform (Thermo Fisher). This study was approved by the Human Ethics Review Committee of Yamanashi University Hospital (Receipt number: 1326 and 1847), and a written informed consent was obtained from all research participants.

### 2.2 Genetic mutational analysis of tissue samples using NGS

The genetic analysis of tumor specimens was performed by amplifying the extracted DNA (10 ng) using barcode adaptors (Ion Xpress Barcode Adapters 1–96 Kit, Life Technologies) with the Ion AmpliSeq Cancer Hotspot panel v.2 (Thermo Fisher), which contains 207 primer pairs and targets approximately 2800 hotspot mutations in the following 50 cancer‐related genes from the COSMIC database^9^: *ABL1*, *AKT1*, *ALK*, *APC*, *ATM*, *BRAF*, *CDH1*, *CDKN2A*, *CSF1R*, *CTNNB1*, *EGFR*, *ERBB2*, *ERBB4*, *EZH2*, *FBXW7*, *FGFR1*, *FGFR2*, *FGFR3*, *FLT3*, *GNA11*, *GNAS*, *GNAQ*, *HNF1A*, *HRAS*, *IDH1*, *JAK2*, *JAK3*, *IDH2*, *KDR/VEGFR2*, *KIT*, *KRAS*, *MET*, *MLH1*, *MPL*, *NOTCH1*, *NPM1*, *NRAS*, *PDGFRA*, *PIK3CA*, *PTEN*, *PTPN11*, *RB1*, *RET*, *SMAD4*, *SMARCB1*, *SMO*, *SRC*, *STK11*, *TP53*, and *VHL*. Barcoded libraries were amplified using emulsion PCR on Ion Sphere particles, and sequencing was performed with an Ion Chef System and an Ion Proton Sequencer (Life Technologies) using an Ion PI Hi‐Q Chef Kit (Life Technologies). Research data obtained in this study are not shared.

### 2.3 Identification of gene alterations and suspected microsatellite instability

Gene mutations and CNVs were identified using Ion reporter software version 5.10 (Thermo Fisher). Furthermore, to avoid false‐positive variants due to sequencing errors, only mutations and CNVs with a mutant allele frequency of >4% (with a sequence read depth of >100) and a copy number >6 were considered truly present in the tissues. The number of altered genes was defined as the number of genes with either mutations or CNVs among the 50 cancer‐related genes that were sequenced in this study. The number of mutations included all multiple mutations of the same gene and all additional mutations other than hotspot mutations in the COSMIC database. Detected gene alterations were then matched with the OncoKB,^10^ which is a knowledge base for precision medicine to infer whether any existing molecular targeted drug is predicted to be effective.

Suspected microsatellite instability (sMSI) was detected using the MSIsensor tool,^11^ which uses the C++ program to compute the length distributions of microsatellites per site from sequence reads. The Ion AmpliSeq Cancer Hotspot panel v.2 contains four microsatellite sites in its target region, and sMSI was defined as no less than one abnormality in the length distributions of microsatellite among the four microsatellite sites.

### 2.4 immunohistochemistry (IHC) of the mismatch repair (MMR) genes

Anti‐MLH1 antibodies (1:250 dilution; ab92312; Abcam plc), anti‐MSH2 antibodies (1:8000 dilution; ab227941; Abcam plc), anti‐MSH6 antibodies (1:500 dilution; ab92471; Abcam plc), and anti‐PMS2 antibodies (1:100 dilution; ab110638; Abcam plc) were used as the primary antibodies (Figure S1). IHC staining was performed according to the manufacturer's instructions. Briefly, 3‐μm‐thick deparaffinized sections of FFPE were stained with the primary antibodies specific for the above MMR genes. Antigens were retrieved by boiling tissue sections in Target Retrieval Solution (Dako). Envision+Dual Link HRP (Dako) was used as the secondary antibody, and diaminobenzidine was used as the chromogen. IHC staining was blindly examined by two independent investigators.

### 2.5 Statistical analysis

Factors associated with overall survival were identified by a Cox multivariate regression analysis, in which the hazard ratio (HR) was adjusted by age and gender and was considered significant when *p* < 0.05. Given the small sample size, Cox regression analysis was repeated multiple times and each iteration had only three factors. Of these, two were constant, namely, age and gender, and the third included variables such as location, size, therapy, etc. All statistical analyses of recorded data and graphic creations were performed using the Lifelines program (https://zenodo.org/badge/latestdoi/12420595) with the Python platform.

## 3 RESULTS

### 3.1 Patient characteristics and qualitative assessments of extracted DNA and NGS

Table 1 shows the clinical characteristics of the 58 patients, among whom 37 (64%) were in stage IV and 48 (83%) had received chemotherapy. The tissue samples were obtained by EUS‐FNA or endoscopic biopsy in 50 (86%) and 8 (14%) cases, respectively. A histological diagnosis of tubular adenocarcinoma was made in 42 (72%) patients; conversely, 11 (19%) patients who were diagnosed with malignancy by cytological testing during EUS‐FNA, endoscopic retrograde cholangiopancreatography (ERCP), or percutaneous transhepatic biliary drainage could not be diagnosed histologically.

**TABLE 1:** Patient characteristics

		PDC (N = 58)
Age	Median (range)	68.5 (44–86)
Sex	Male/Female	34/24
PDC location	Ph/Pbt	30/28
PDC size (mm)	Median (range)	34.5 (12–70)
PDC stage	II/III/IV	16/5/37
Therapy	Operation/CRT/Chemo/BSC	6/1/48/3
Procedure	EUS‐FNA/Duodenal biopsy	50/8
Histology^a^	tub	42
	tub/sq	2
	por/sig	3
	diagnosed as malignancy only by cytology	11

Abbreviations: BSC, best supportive care; Chemo, chemotherapy; CRT, chemoradiotherapy; EUS‐FNA, endoscopic ultrasound‐guided fine needle aspiration; Pbt, pancreatic body and tail; PDC, Pancreatic ductal carcinoma; Ph, pancreatic head; por/sig, poorly differentiated adenocarcinoma with signet cell component; tub, adenocarcinoma; tub/sq, tub. with squamous component.

^a^ Initial histological diagnosis.

The average (±standard deviation [SD]) and median (range) quantities of the extracted DNA from the FFPE samples obtained by EUS‐FNA or endoscopic biopsy were 42.9 ng (±53.3) and 19.2 ng (3.8–228), respectively, except for 11 samples that had DNA concentrations below the limit of detection (LOD). In the NGS analyses of tissues obtained by EUS‐FNA, the target regions of the 50 cancer‐related genes included 22,027 bases, and the average (±SD) and median (range) sequenced read depths were 4912 (±3516) and 4,008 (1164–19,798), respectively. The yield of extracted DNA (median, range) was lower in EUS‐FNA samples compared to that from endoscopic biopsy [18 ng (3.8–136) vs. 133 ng (24–228), respectively; *p* = 0.041], whereas the number of samples with mutation in any gene was not different between these two sets of samples [46/50 (82%), EUS‐FNA vs. 8/8 (100%), duodenal biopsy; *p* = 0.938], even though duodenal biopsy samples tended to have more mutations (Table S2).

### 3.2 Frequently altered and targetable genes in pancreatic cancer

**FIGURE 1:**
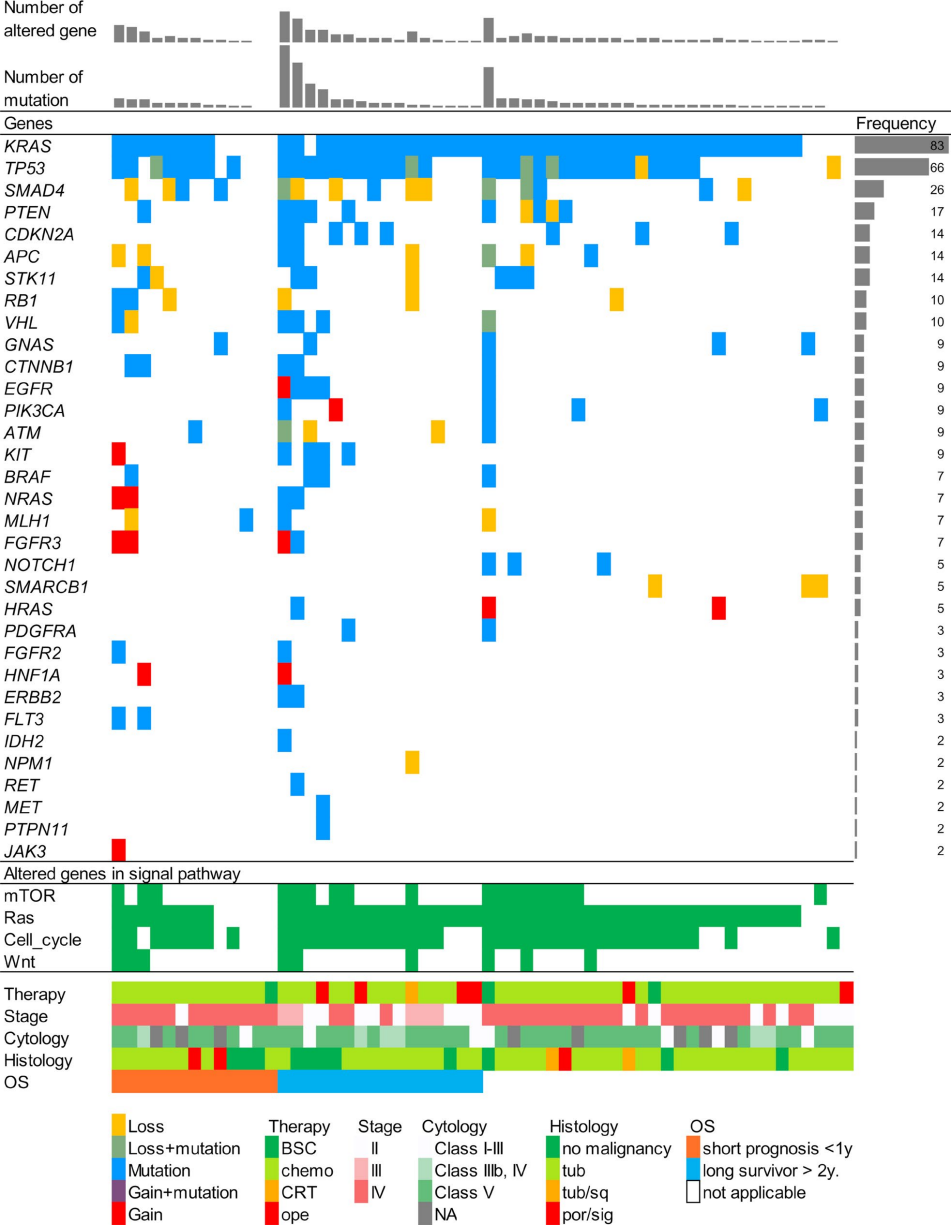
Gene alterations and clinical characteristics of pancreatic cancer. The panel shows the overall view of the detected gene alterations in tissues from endoscopically obtained pancreatic cancer specimens. The boxes in the center panel represent detected gene alterations and altered genes in signaling pathways in each case. The left side of the panel shows gene symbols, and the frequencies of mutations in each gene are shown in the right side of the panel. The bar graphs on the upper side of the panel show the number of altered genes and the number of mutations in each case. The lower side of the panel shows the color indicators and clinical characteristics of each case

Pancreatic cancer gene alterations in the endoscopically obtained tissue samples were identified for 33 of the 50 analyzed cancer‐related genes, and alterations of any type were detected in 55 cases (94.8%, Figure 1). Furthermore, some type of gene alteration was detected in all samples with extracted DNA concentrations below the LOD. The four most frequently altered genes in the tissue samples were *KRAS* (83%), *TP53* (66%), *SMAD4* (26%), and *PTEN* (17%), followed by *CDKN2A* (14%), *APC* (14%), and *STK11* (14%). Five (8.6%) and 10 (17.2%) patients had samples with more than 10 mutations and more than five altered genes, respectively. When summarized by signaling pathway, the mTOR (*FLT3*, *PTEN*, *STK11*, *PIK3CA*, and *AKT1*), Ras (*ERBB4*, *EGFR*, *HRAS*, *NRAS*, *KRAS*, *PDGFRA*, *KIT*, *FGFR3*, *ERBB2*, *BRAF*, *MET*, *FGFR1*, and *FGFR2*), cell cycle (*ATM*, *RB1*, *TP53*, and *CDKN2A*), and Wnt (*APC* and *CTNNB1*) signaling pathways were activated by their constituent gene alterations in 31%, 84%, 69%, and 16% of samples, respectively. We next matched detected gene alterations with the OncoKB, which classifies genetic alterations into four levels according to an actionability scale: levels 1–3A indicate standard therapeutic intervention or compelling clinical evidence for the disease, level 3B indicates the presence of clinical evidence for another disease, and level 4 indicates the presence of compelling biological evidence. Although none of the detected gene alterations corresponded to levels 1–3A of the OncoKB, 20 gene alterations in 13 cases (22.4%) corresponded to level 3B, including alterations in *ATM*, *NRAS*, *ERBB2*, *PIK3CA*, *KIT*, and *IDH2* (Table S3).

### 3.3 Genetic and clinical factors associated with overall survival

**FIGURE 2:**
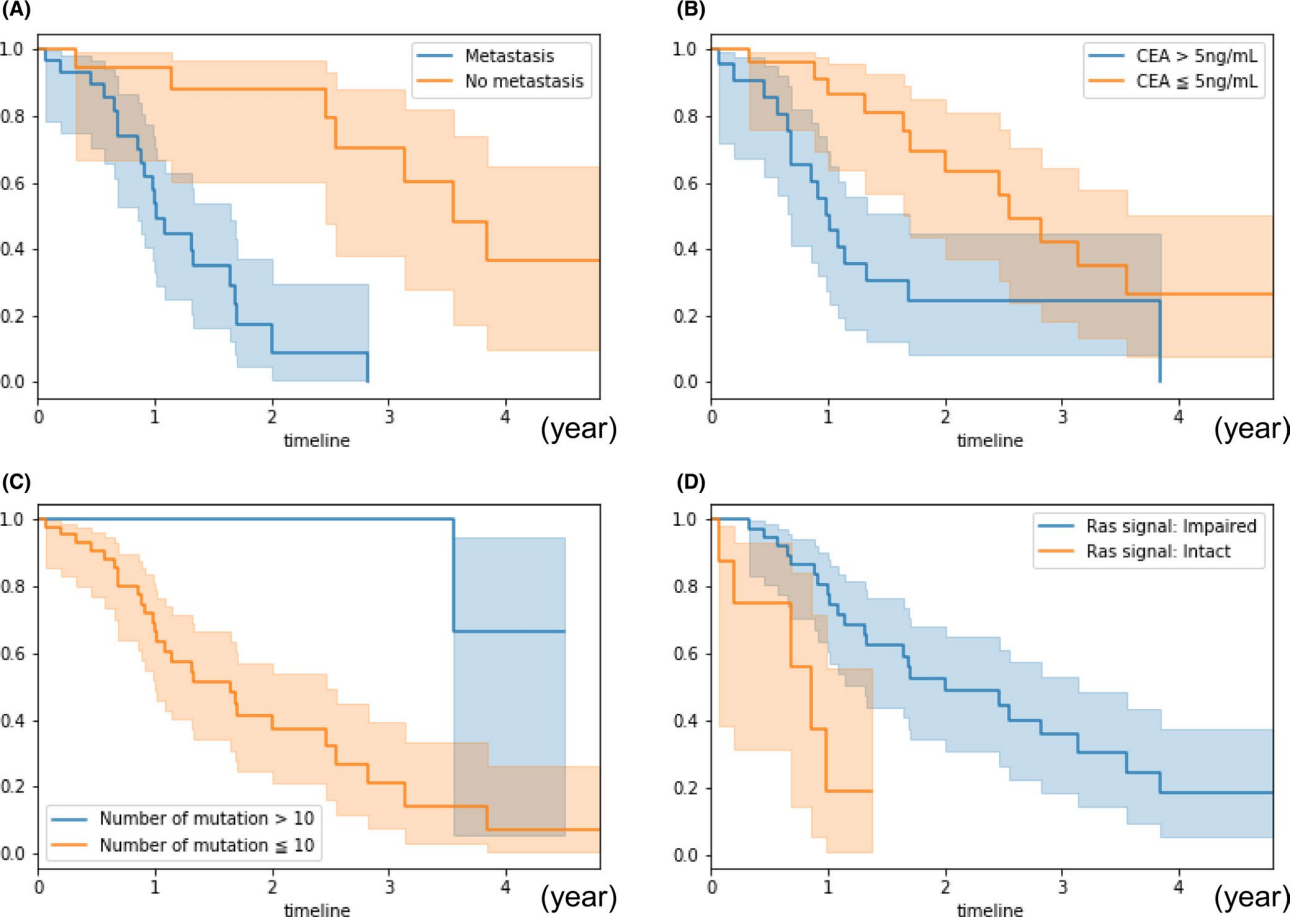
Immunohistochemistry for mismatch repair genes in endoscopically obtained samples. The immunohistochemistry and hematoxylin and eosin staining of four mismatch repair genes, MLH1, PMS2, MSH2, and MSH6, are shown. Images of cases 4 and 7 correspond to the cases with a high number of mutations and a control case, in which all four MMR genes were stained, in Table 3

Because the relationship between genetic factors and clinical factors including overall survival remains poorly understood, we performed a Cox regression analysis for overall survival with genetic and clinical factors by adjusting for age and gender (Table 2). The analysis revealed that the presence of metastasis (*p* < 0.005, HR 10.1), serum CEA levels >5 ng/mL (*p* = 0.01, HR 2.86), ≤10 detected hotspot mutations (*p* = 0.03, HR 9.86), and intact Ras signaling (*p* < 0.005, HR 5.57) were risk factors for a shorter prognosis (Figure 2). We also included MSI status calculated by the MSIsensor program using sequence reads in this overall survival analysis; MSI status had no relationship with overall survival (*p* = 0.10, Table 2) or the number of mutations.

**TABLE 2:** Cox regression analysis for survival adjusted by age and gender

Characteristics		N (total = 58)	Adjusted HR (95% CI)^a^	*p*
Location	Ph	30	1.03 (0.45–2.33)	0.94
Size	>20 mm	50	1.77 (0.41–7.55)	0.44
Therapy	Non‐operation	52	6.13 (0.79–47.5)	0.08
Metastasis	Present	37	10.1 (3.15–32.4)	<0.005*
CEA	>5 ng/ml	30	2.86 (1.30–6.28)	0.01*
CA19‐9	>100 U/ml	31	1.57 (0.73–3.41)	0.25
MSI status	sMSI	2	3.71 (0.79–17.4)	0.10
Number of mutations	≤10	53	9.86 (1.21–80.1)	0.03*
Number of altered genes	≤5	48	2.28 (0.71–7.35)	0.17
*KRAS*	WT	10	2.49 (0.87–7.09)	0.09
*TP53*	WT	20	1.76 (0.81–3.82)	0.15
*SMAD4*	WT	43	1.09 (0.47–2.52)	0.84
*PTEN*	WT	48	1.76 (0.59–5.23)	0.31
*CDKN2A*	WT	50	1.89 (0.64–5.64)	0.25
*APC*	WT	50	1.57 (0.45–5.48)	0.48
*STK11*	WT	50	2.44 (0.55–10.9)	0.24
*VHL*	WT	52	1.87 (0.50–6.97)	0.35
*RB1*	WT	52	0.58 (0.18–1.82)	0.35
mTOR signaling^b^	Intact	40	1.31 (0.53–3.19)	0.56
Ras signaling^c^	Intact	9	5.57 (1.80–17.3)	<0.005*
Cell cycle signaling^d^	Intact	18	1.67 (0.73–3.80)	0.22
Wnt signaling^e^	Intact	49	1.14 (0.37–3.47)	0.82

Abbreviations: Ph, pancreatic head; sMSI, suspected microsatellite instability; WT, wild type.

Intact, no mutation in related genes.

^a^ Hazard ratio adjusted by age and gender with 95% confidence intervals.

^b^ *FLT3*, *PTEN*, *STK11*, *PIK3CA*, *AKT1*.

^c^ *ERBB4*, *EGFR*, *HRAS*, *NRAS*, *KRAS*, *PDGFRA*, *KIT*, *FGFR3*, *ERBB2*, *BRAF,* *MET,* *FGFR1,* *FGFR2*.

^d^ *ATM*, *RB1*, *TP53*, *CDKN2A*.

^e^ *APC*, *CTNNB1*.

* *p* < 0.05.

### 3.4 Detailed analysis of cases with a high number of mutations and suspected MSI

**FIGURE 3:**
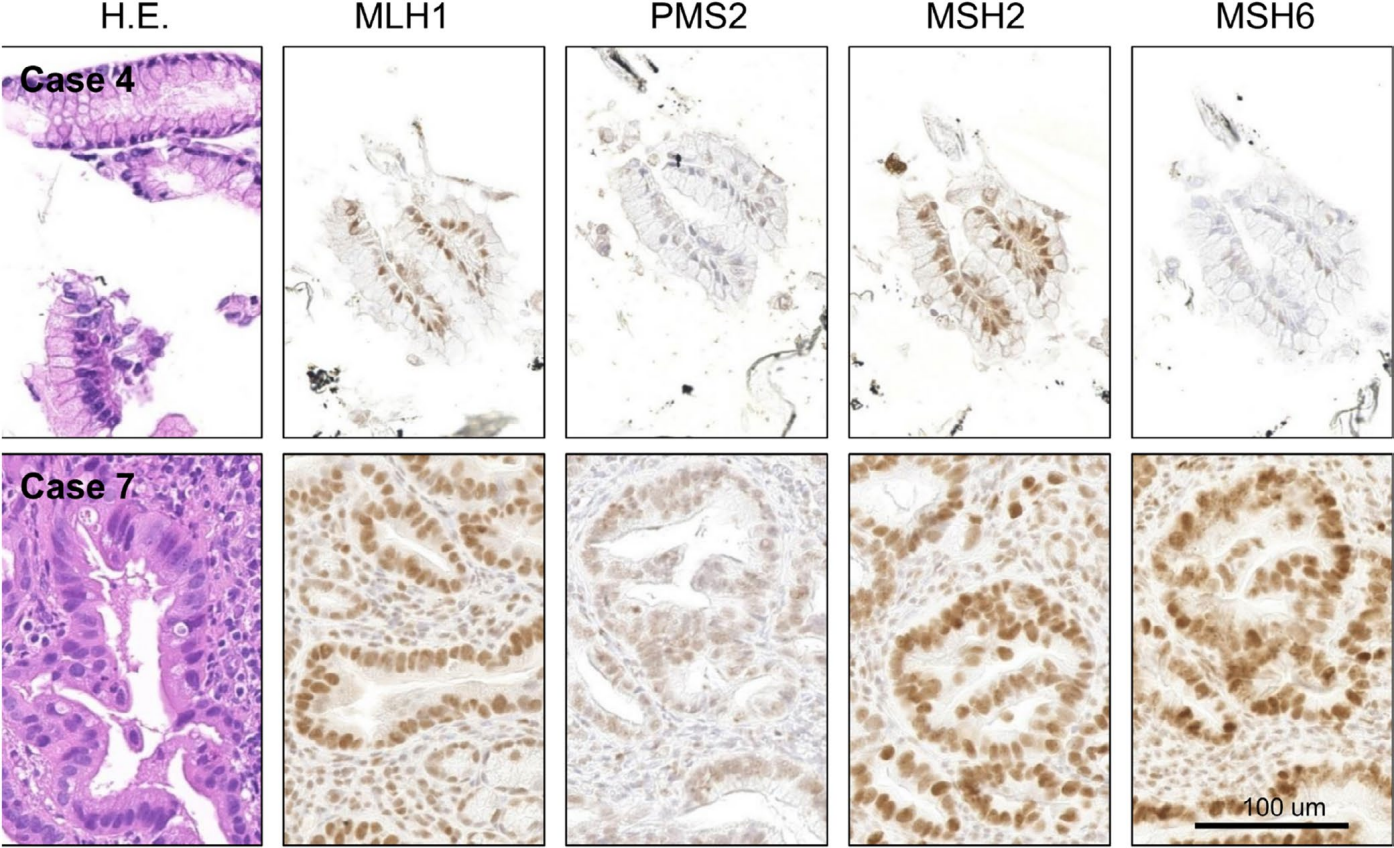
Different overall survival rates due to clinical and genetic factors. Overall survival curves stratified by four the clinical and genetic factors that displayed statistical significance in the Cox proportional hazards model. a Presence or absence of metastasis. b Serum CEA levels. c Number of mutations detected by the analysis of 50 cancer‐related genes. d Intact or impaired Ras signal. The Ras signal is composed of *ERBB4*, *EGFR*, *HRAS*, *NRAS*, *KRAS*, *PDGFRA*, *KIT*, *FGFR3*, *ERBB2*, *BRAF*, *MET*, *FGFR1*, and *FGFR2* in this analysis

Given the lack of an association between sMSI and the number of mutations, we presented detailed genetic and clinical data from cases with either sMSI, a higher number of mutations, or *MLH1* alterations (Table 3), along with the IHC results of MMR genes by staining for MLH1, PMS2, MSH2, and MSH6 (Figure 3). Mutations in MLH1 or MSH2 are reported to result in the concurrent loss of MLH1/PMS2 or MSH2/MSH6, respectively, by IHC, whereas mutations in PSM2 or MSH6 result in the isolated loss of PMS2 or MSH6^12^; consequently, five cases (excluding cases 2, 7, and 8) in Table 3 were identified MMR‐deficient tumors. Although MSI pancreatic cancers are reported to be associated with *KRAS*‐*TP53* wild type and *JAK* gene mutations,^13^ only one case was *KRAS*‐*TP53* wild type, and no *JAK* mutations were found among the eight cases listed in Table 3.

**TABLE 3:** Immunostaining of mismatch repair genes in cases with high number of mutation or with sMSI

Case	Age	Gender	Stage	Number of mutation	MSIsensor	Gene mutation					Immunohistochemistry	
						*KRAS*	*TP53*	*GNAS*	*MLH1*	*JAK2/3*	MLH1/PMS2	MSH2/MSH6
1	61	Male	III	40	N.D.	G12R	G244S	WT	V384D	WT	+/−	+/−
2	71	Male	III	29	N.D.	G12D	E294K	WT	WT	WT	N.A.	N.A.
3	83	Male	IV	26	N.D.	G12R	R342*	R201C	CN loss	WT	+/−	+/+
4	67	Male	II	15	N.D.	WT	E271K	R201C	WT	WT	+/−	+/−
5	62	Male	II	12	N.D.	G12D	P72A	WT	WT	WT	+/−	+/−
6	69	Male	IV	5	sMSI	G12D	S95F	WT	CN loss	WT	+/−	+/+
7	57	Female	IV	2	sMSI	G12V	WT	WT	WT	WT	+/+	+/+
8	78	Male	IV	1	N.D.	WT	WT	WT	V384D	WT	N.A.	N.A.

Note

N.D., MSI was not detected by MSIsensor.

Abbreviations: +, present; −, absent; *, stop codon; CN, copy number; N.A., not available; sMSI, suspected microsatellite instability; WT, wild type.

**FIGURE 4:**
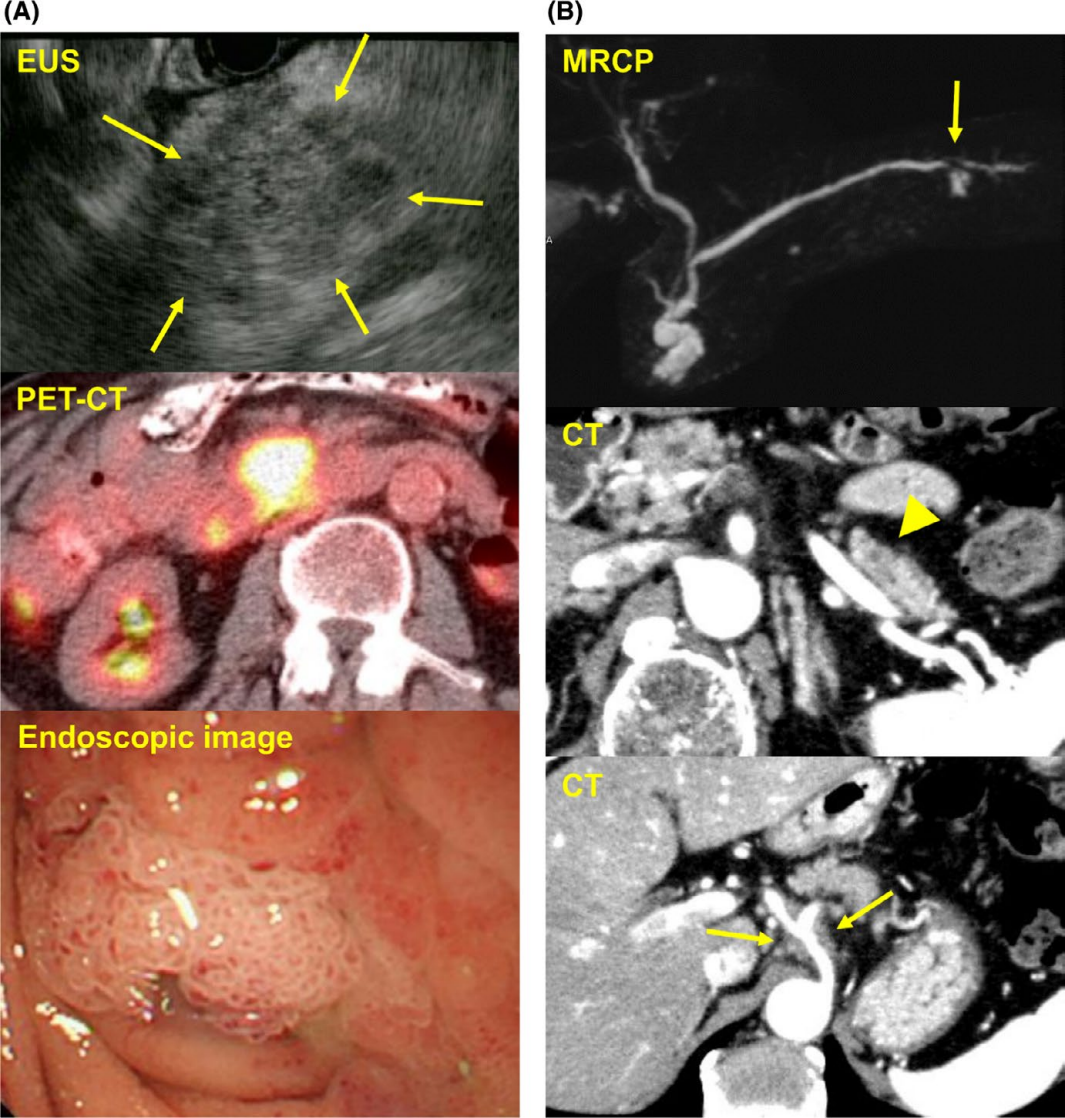
Clinical images of cases with a high number of mutations together with the *GNAS* mutation. Clinical images of cases with the GNAS mutation in Table 3 are shown. (a) Case 3 in Table 3. Positron Emission Tomography (PET)‐CT shows the accumulation of injected agents in the pancreas head in which a hypoechoic tumor with cystic lesions was observed by endoscopic ultrasonography (EUS, yellow arrows). Endoscopic image showing that the tumor invaded the duodenal lumen. (b) Case 4 in Table 3. Magnetic resonance cholangiopancreatography (MRCP) shows cystic lesions in the pancreas head and tail (yellow arrow). CT shows a hypo‐enhanced mass with a cystic lesion in the pancreas tail (yellow arrowhead) and shows the tumor extending along the arteries (yellow arrow)

A mutation in *GNAS* was found in two cases (cases 3 and 4 in Table 3), and thus, these cases were presumed to be intraductal papillary mucinous neoplasms (IPMNs). Clinical images show that a solid tumor with a cystic component and a papillary morphology in the endoscopic view invaded the duodenum (case 3 in Table 3; Figure 4a) and that a tumor with a cystic lesion identified by MRCP extended to the surrounding splenic and celiac arteries (case 4, Figure 4b); both of these cases could be diagnosed as IPMNs by clinical images.

## 4 DISCUSSION

In this study, we performed an NGS analysis of endoscopically obtained pancreatic cancer tissue samples using the compact gene panel, which can examine 50 cancer‐related genes. By using the compact gene panel, NGS analysis could detect actionable genes, cases with more mutations, and sMSI, which can be used to determine the efficacy of immune checkpoint inhibitors and to identify prognostic factors in a cost‐effective manner and short time frame.

To apply precision medicine for pancreatic cancer, genetic analyses must be performed in a short time even with the small samples that can be clinically obtained. Furthermore, abundant interstitial pancreatic cancer tissue can hinder the detection of gene alterations by extensive gene analysis with insufficient sequence depths, particularly when the tissue samples are tiny. Therefore, targeted sequencing of endoscopically obtained FFPE samples using a compact panel with 50 cancer‐related genes, as performed in this study, is significant for actual clinical practice. Recent whole‐exome and/or whole‐genome sequencing analyses of pancreatic cancer have revealed that *KRAS* (65%–95%), *TP53* (33%–66%), *SMAD4* (16%–23%), and *CDKN2A* (19%–20%) were the most frequently altered genes, followed by *TTN* (12%–16%).^4^, ^5^, ^14^, ^15^ These findings are consistent with our results in the detectability by compact sequencing; however, *TTN* was not included in the panel we used. Although the detection rate of *KRAS* alterations in our study was somewhat lower (83%) than that reported in another extensive targeted sequence analysis (95%)^16^ or in a whole‐exome sequence analysis (95%),^14^ these studies selected samples with enough tumor cellularity or concentrated tumor cells from tumor xenografts. The *KRAS* alteration rate in our study tended to be lower in histologically negative samples than in positive samples (64% vs. 87%, *p* = 0.08), which seemed to reflect data from samples available in real practice.

Targeted sequencing of compact range of cancer‐related genes was helpful in predicting prognosis and selecting therapy. Recent comprehensive genetic analyses have uncovered whole genetic abnormalities of pancreatic cancer and their contribution to its carcinogenesis. For example, whole‐exome sequencing uncovered novel additional mutated genes involved in chromatin modification, DNA damage repair, and SLIT/ROBO signaling that are known as embryonic regulators of axon guidance genes in addition to previously known mutations^14^; moreover, whole‐genome sequencing revealed that cases with defective DNA maintenance could respond to platinum therapy.^4^ Furthermore, targeted sequencing with cancer‐related genes revealed the relationships between the altered gene and metastatic sites, age, gender, smoking, etc.,^16^, ^17^ and alterations in *GNAS* and *RB1* were reported to be associated with disease recurrence.^16^ Here, we show an association between prognosis and genetic mutations identified by targeted sequencing of 50 cancer‐related genes and also reveal that a poor prognosis is associated with fewer gene mutations, intact Ras signaling, presence of metastasis, and elevated serum CEA levels.

The combination of counting the number of mutations per sample and using an existing algorithm to calculate MSI could identify cases with MSI with the use of the compact cancer‐related gene panel. Defective MMR genes and a subsequent increasing tumor mutational burden (TMB) and MSI are associated with the response to newly developed immune checkpoint inhibitors^18^, ^19^; however, MSI is very rare in pancreatic cancer and occurs in approximately 0.8–2% of cases.^13^, ^20^, ^21^ The TMB has become a useful marker for predicting the presence of defective MMR and is usually defined as high when a tumor contains ≥12 somatic mutations per megabase.^22^ The panel we used in this study was as small as 22 kilobases; thus, the precise TMB could not be calculated. Therefore, we calculated the number of mutations within the 22 kb panel and used this value as an alternative mutational burden (MB). Another method to predict the presence of defective MMR is to detect MSI, which can be calculated using sequence reads by computing algorithms such as MSIsensor.^11^ Because both the MSIsensor and TMB calculations were slightly disadvantageous for a small gene panel, we combined both methods to screen for defective MMR and confirmed our findings by IHC. Seven of the 58 cases were screened by these methods, and five (8.6%) were confirmed as defective MMR by IHC. The percentage of defective MMR cases was larger in our cohort than in previous reports partly because our cohort included a larger proportion of patients with advanced pancreatic cancer and cancers associated with IPMNs. Previous studies have reported that a higher proportion of advanced^23^ or IPMN‐associated pancreatic cancers^24^ had MSI.

Uniquely, we show a relationship between clinical factors and genetic mutations in EUS‐FNA samples from a cohort of predominantly unresectable pancreatic tumors. Notably, while our cohort included mainly unresectable pancreatic cancers, except for a few reports,^16^ published data on genetic analysis in pancreatic cancer have used resected tumor samples. Thus, we think this study will be valuable in understanding the relationship between genetic alterations and features of unresectable pancreatic tumors as our analysis revealed that the poor prognosis was not only unquestionably related to metastasis and high CEA value, but also to a high number of mutations, and intact Ras signaling. The relationship between improved survival and high MB has been controversial in pancreatic cancer probably due to the low prevalence of cases with high MB.^20^ In contrast, improved survival has been reported in colorectal cancer with high MB, by studies with large sample sizes.^25^ Additionally, although there are few pancreatic cancer cases with high MB, IPMN with high MB are relatively more prevalent.^24^ We think the reason for cases with high number of mutations showing improved survival is due to our study cohort comprising predominantly unresectable pancreatic cancers, thereby unexpectedly including a certain number of IPMN‐derived pancreatic cancers. Cases with intact Ras signaling in our cohort showed poor prognosis; however, it must be noted that the relation between Ras signaling and prognosis is controversial. First, even though a meta‐analysis of *KRAS* mutations in pancreatic cancer reported poorer prognosis,^26^ the cohorts used in the meta‐analyses included fewer cases with *KRAS* mutation (60%–70% cases) compared to other studies that usually report a 90% prevalence of *KRAS* mutations. Second, other reports have stated that cases with *KRAS* mutations, especially in codon Q61 alleles, have a better prognosis compared to others by whole‐exome sequencing analysis.^25^ Third, basic research reports that have used pancreatic cancer cells wherein *KRAS* function had been inhibited by CRISPR/Cas show that these cells not only activate phosphoinositide 3‐kinase (PI3K)‐dependent mitogen‐activated protein kinase signaling, but also induce metastasis‐related cascade, including EMT (*TGFB2*, *PBX1*, and *FGFBP1*), cell adhesion (*FLRT3* and *ICAM1*), and extracellular matrix breakdown (*MMP19* and *MMP28*).^27^ Collectively, a survival effect of Ras signaling on pancreatic cancer may be plausible, but remains questionable and these factors may have affected the results from our cohort of unresectable pancreatic cancer.

Multiple clinical implications are fostered by the findings of this study. First, our analysis conquered the disadvantages in obtaining pancreatic cancer samples. Outsourced genomic analyses require approximately 200 ng of DNA or 1.25 mm^3^ of tissue, which are not easily obtainable in real practice. In our study, we performed an NGS analysis that provided useful information using a maximum of 0.28 mm^3^ of FFPE tissues and a median of 19.2 ng of DNA. Moreover, some type of gene alteration was detected in all 11 cases with DNA concentrations below than the LOD. Second, important information regarding prognosis and treatment selection can be obtained by a simple gene analysis.

This study has several limitations. First, the design is retrospective, and hence, only a small number of cases were recruited from a single center. Second, as mentioned above, using a small gene panel is disadvantageous for calculating TMB and MSI; therefore, we confirmed our results by IHC, renamed our analyses as “sMSI” (replacing MSI), and changed tumor mutational burden to number of mutations. We considered the use of sMSI, the number of mutations, and MLH1 alterations for MMR deficiency screening to be appropriate and confirmed the results by IHC. Third, among the 58 cases included in this study, four cases tested negative for EUS‐FNA‐based diagnosis. Of these, two were diagnosed based on cytology of ascites and bile juice, while the other two were identified by imaging and their malignant clinical course. These pathology‐negative cases also had gene alterations in *KRAS*, *TP53*, *SMAD4*, and others, which is consistent with pancreatic cancer (Table S1).

In conclusion, we performed small but targeted sequencing of endoscopically obtained pancreatic cancer FFPE samples that were available from real clinical practice and evaluated the relationship between gene alterations and prognosis to help determine treatment choices. We believe that these findings will improve the clinical outcomes of pancreatic cancer.

## CONFLICT OF INTEREST

The authors disclose no potential conflicts of interest.

## AUTHOR CONTRIBUTIONS

All authors contributed to the study conception and design. Material preparation, data collection, and data analyses were performed by Mitsuharu Fukasawa, Hiroko Shindo, Ei Takahashi, Sumio Hirose, Yoshimitsu Fukasawa, Satoshi Kawakami, Hiroshi Hayakawa, Natsuhiko Kuratomi, Makoto Kadokura, Shinya Maekawa, Tadashi Sato, and Nobuyuki Enomoto. The first draft of the manuscript was written by Shinichi Takano, and all authors commented on previous versions of the manuscript. All authors read and approved the final manuscript.

## Supporting information

Fig S1

Fig S1_1

Table S1‐3

Table S1‐3_1
