# Supplementary figures and images for: Clinical significance of genetic alterations in endoscopically obtained pancreatic cancer specimens
Source: Cancer Med. 2021 Jan 16;10(4):1264–74. doi: 10.1002/cam4.3723 (PMC7926030; doi:10.1002/cam4.3723)

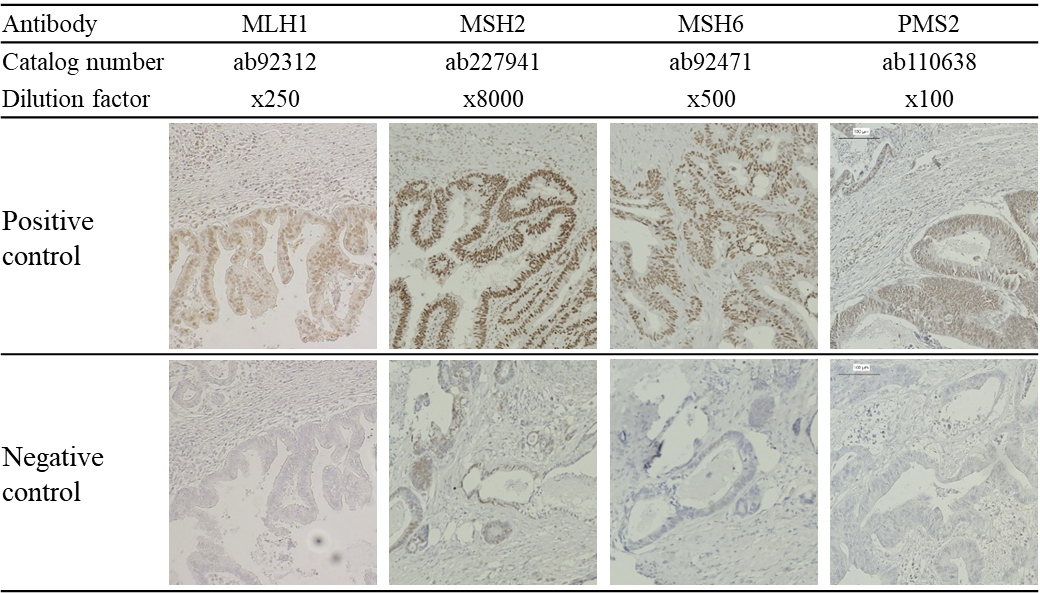

Supplement: Supplementary file 1 — Fig S1 [file CAM4-10-1264-s001.docx]
