# Supplementary material for: Clinical significance of genetic alterations in endoscopically obtained pancreatic cancer specimens
Source: Cancer Med. 2021 Jan 16;10(4):1264–74. doi: 10.1002/cam4.3723 (PMC7926030; doi:10.1002/cam4.3723)
Supplement: Supplementary file 3 — Table S1‐3 [file CAM4-10-1264-s003.docx]

| Table S1. Characteristics of cases that were pathologically negative for EUS-FNA. | | | | | | | | |  |  |  |  |
| --- | --- | --- | --- | --- | --- | --- | --- | --- | --- | --- | --- | --- |
|  |  |  |  |  |  | EUS-FNA | |  |  |  |  |  |
| Age | Gender | Location | Stage | CEA (ng/mL) | CA19-9 (U/ml) | Cytology | Histology | Therapy | Number of mutations | Number of altered genes | Gene alterations (up to 5) | Basis of clinical diagnosis |
| 78 | Male | Pt | IV | 7.6 | 603.1 | Class I | No malignancy | Chemotherapy | 1 | 1 | *MLH1* | Ascites cytology |
| 44 | Male | Ph | IV | 3.8 | 1723 | Class III | Atypical epithelium | Chemotherapy | 2 | 2 | *KRAS, TP53* | Imaging and clinical course |
| 67 | Male | Pt | II | 1.5 | 6.1 | Class III | Atypical epithelium | Chemotherapy | 15 | 8 | *TP53, PTEN, STK11, GNAS, EGFR* | Bile cytology |
| 83 | Male | Ph | IV | 8.6 | 158 | Class III | Atypical epithelium | BSC | 26 | 16 | *KRAS, TP53, SMAD4, PTEN, GNAS* | Imaging and clinical course |

| Table S2. DNA quantity and coverage analyses of NGS | | | |  |
| --- | --- | --- | --- | --- |
|  |  | EUS-FNA | Duodenal biopsy |  |
|  |  | (N = 50) | (N = 8) | *P* |
| DNA quantity from biopsy sample | |  |  |  |
|  | Number of samples below LOD* | 11 (19%) | 0 (0%) | 0.323 |
|  | Median (range), ng† | 18 (3.8–136) | 133 (24–228) | 0.041 |
|  | Average (SD), ng† | 26.7 (29.8) | 122 (72.3) | <0.001 |
|  |  |  |  |  |
| Sequence read depth | |  |  |  |
|  | Median (range) | 4,653 (1,164–19,798) | 1,907 (1,212–4,462) | 0.009 |
|  | Average (SD) | 5,310 (3,607) | 2,426 (1,191) | 0.030 |
|  |  |  |  |  |
| Number of samples with mutation | |  |  |  |
|  | *KRAS*, n (%) | 41 (82%) | 7 (87.5%) | 0.903 |
|  | Any gene, n (%) | 46 (92%) | 8 (100%) | 0.938 |
| LOD*, concentration below limit of detection; | | | | |
| †, median or average among samples other than LOD | | | | |

| Table S3. Actionable gene alterations detected by the knowledge database | | | | |
| --- | --- | --- | --- | --- |
| Case | Gene symbol | Mutation | OncoKB level | Responsible molecular targeted drugs |
| 1 | *ATM* | p.R3008C | 3B | Olaparib |
| 1 | *NRAS* | p.G13S | 3B | Binimetinib, Binimetinib+Ribociclib |
| 1 | *ERBB2* | p.G776S | 3B | Ado-Trastuzumab Emtansine, Neratinib |
| 1 | *PIK3CA* | p.E453K | 3B | Fulvestrant+Alpelisib, GDC-0077, Copanlisib+Fulvestrant |
| 1 | *PIK3CA* | p.E542K | 3B | Fulvestrant+Alpelisib, GDC-0077, Copanlisib+Fulvestrant |
| 2 | *NRAS* | p.G12S | 3B | Binimetinib, Binimetinib+Ribociclib |
| 3 | *ATM* | p.R3008C | 3B | Olaparib |
| 3 | *NRAS* | p.G12D | 3B | Binimetinib, Binimetinib+Ribociclib |
| 4 | *ATM* | p.I2888T | 3B | Olaparib |
| 5 | *KIT* | p.P573L | 3B | Imatinib |
| 8 | *ATM* | p.R337C | 3B | Olaparib |
| 9 | *ATM* | p.R3008H | 3B | Olaparib |
| 10 | *IDH2* | p.R172K | 3B | Enasidenib |
| 10 | *KIT* | p.P573L | 3B | Imatinib |
| 11 | *ERBB2* | p.V777M | 3B | Ado-Trastuzumab Emtansine, Neratinib |
| 12 | *NRAS* | p.G13D | 3B | Binimetinib, Binimetinib+Ribociclib |
| 13 | *PIK3CA* | p.E545K | 3B | Fulvestrant+Alpelisib, GDC-0077, Copanlisib+Fulvestrant |
| 14 | *ERBB2* | p.V777M | 3B | Ado-Trastuzumab Emtansine, Neratinib |
| 14 | *ERBB2* | p.V842I | 3B | Ado-Trastuzumab Emtansine, Neratinib |
| 15 | *ATM* | p.R3008H | 3B | Olaparib |
